# Vitamin D and L-Cysteine as Potential Regulators of Adiponectin in Alzheimer’s Disease: A Narrative Review

**DOI:** 10.3390/nu18152440

**Published:** 2026-07-26

**Authors:** Jeffrey Justin Margret, Sushil K. Jain

**Affiliations:** Department of Pediatrics, Louisiana State University Health Shreveport, Shreveport, LA 71103, USA; jeffrey.justinmargret@lsuhs.edu

**Keywords:** adiponectin, Alzheimer’s disease, glutathione, L-cysteine, Vitamin D

## Abstract

Alzheimer’s disease (AD) is a progressive neurodegenerative disorder marked by amyloid-β buildup, tau pathology, neuroinflammation, and declining cognition. Adiponectin, a hormone produced by adipose tissue with insulin-sensitizing, anti-inflammatory, and antioxidant effects, may link peripheral metabolic health to brain function. Studies show that adiponectin helps neurons survive, improves synaptic plasticity, maintains blood–brain barrier integrity, and reduces amyloid-β and tau damage via AdipoR1/R2 signaling. Clinical studies evaluating circulating adiponectin have yielded inconsistent findings, giving rise to the ‘adiponectin paradox’ whereby elevated adiponectin levels in older adults and patients with AD may reflect frailty, weight loss, systemic inflammation, or compensatory responses rather than direct neuroprotective effects. Studies indicate that vitamin D (VD) and L-cysteine (L-Cys), a precursor to glutathione, act synergistically to modulate oxidative stress, inflammation, and adiponectin levels. VD increases circulating adiponectin and benefits metabolism, while L-Cys boosts glutathione, restores redox balance, and promotes adiponectin secretion by affecting fat cell function. Emerging experimental evidence, together with clinical observations from metabolic disorders, suggests that VD and L-Cys may influence adiponectin-related pathways, oxidative stress, and inflammation, which are implicated in AD pathogenesis. However, direct clinical evidence demonstrating that combined VD and L-Cys supplementation modulates these pathways or alters AD progression in humans is currently lacking. This narrative review covers current knowledge of adiponectin and its links to obesity, metabolic issues, and AD, and it also explores the roles of VD and L-Cys as regulators of adiponectin signaling. Overall, the available evidence supports the proposed interaction among VD–L-Cys and adiponectin, which warrants further mechanistic investigation and well-designed clinical studies to determine its therapeutic relevance in AD.

## 1. Introduction

Alzheimer’s disease (AD) is a progressive neurodegenerative disorder marked by extracellular amyloid-β (Aβ) deposition and intracellular neurofibrillary tangles composed of hyperphosphorylated tau. It leads to synaptic failure and, ultimately, cognitive decline [1,2]. AD is a central nervous system disorder predominantly impacting individuals aged 65 and older. Over 5 million people in the United States currently have a diagnosis of this condition, with mortality rates rising by 89% since the year 2000 [3,4]. Around 50 million individuals worldwide are affected by dementia, and it is estimated that the number will increase threefold by the year 2050 [5]. Although advancing age and genetic susceptibility, most notably the APOE ε4 allele, remain the predominant determinants of AD risk, growing evidence indicates that metabolic dysregulation plays a crucial role in shaping disease onset and progression [6,7]. Within this metabolic framework, the adipokine adiponectin has emerged as a biologically compelling factor linking peripheral metabolic health to central neurodegenerative pathways [8].

Recent studies have highlighted a potential biological interaction between vitamin D (VD) and L-cysteine (L-Cys), two metabolic regulators that converge on glutathione (GSH) homeostasis and VD activation pathways. Co-supplementation with VD and L-Cys has been reported to increase circulating adiponectin levels, with their combined antioxidant and anti-inflammatory effects positioning them as plausible modulators of the adiponectin-related metabolic and neuroinflammatory pathways [9,10,11]. Together, these findings suggest a mechanistic framework in which VD and L-Cys act synergistically to regulate adiponectin, redox balance, and neuroinflammatory pathways, which may influence biological pathways implicated in AD. This review highlights current evidence supporting the VD–L-Cys–adiponectin triad as a potential metabolic–neuroprotective axis and explores its relevance to AD pathophysiology and future therapeutic research.

Although accumulating experimental evidence supports the neuroprotective functions of adiponectin, human studies remain inconsistent and have produced the so-called adiponectin paradox [12]. Furthermore, although VD and L-Cys have independently been shown to influence oxidative stress, glutathione metabolism, and adiponectin expression [10,11], their potential interactions within the context of AD have not been comprehensively synthesized. Addressing these knowledge gaps provides the rationale for the present review.

The objectives of this narrative review are (i) to summarize current knowledge regarding adiponectin biology and its relationship with AD, (ii) to critically evaluate the evidence supporting VD and L-Cys as modulators of adiponectin signaling and metabolic homeostasis, and (iii) to propose a mechanistic framework linking these pathways while highlighting current evidence gaps and future research priorities.

## 2. Methodology

This manuscript comprises a narrative review. A literature search was performed using PubMed and Google Scholar for studies published through January 2005. Search terms included combinations of “adiponectin,” “Alzheimer’s disease,” “dementia,” “vitamin D,” “L-cysteine,” “*N*-acetylcysteine,” “glutathione,” “oxidative stress,” “neuroinflammation,” and “metabolic dysfunction.” Original research articles, clinical trials, systematic reviews, meta-analyses, and relevant mechanistic studies published in English were considered. Studies were selected based on scientific relevance to adiponectin signaling, VD or L-Cys biology, oxidative stress, metabolic regulation, and AD. As this is a narrative review, formal risk-of-bias assessment and quantitative evidence synthesis were not performed.

## 3. Adiponectin

Adiponectin is an adipose-derived endocrine hormone (adipokine) encoded by the *ADIPOQ* gene and synthesized predominantly by white adipose tissue. It plays a central role in maintaining metabolic homeostasis through its insulin-sensitizing, anti-inflammatory, anti-atherogenic, and antioxidant properties. Circulating adiponectin levels are positively associated with insulin sensitivity and regulate energy balance by promoting fatty acid oxidation, reducing triglyceride accumulation, suppressing hepatic gluconeogenesis, and improving glucose utilization [9,13,14]. Women generally exhibit higher circulating adiponectin concentrations than men, partly due to the influence of estrogen on adipose tissue function. Conversely, reduced adiponectin levels have been consistently associated with obesity, insulin resistance, type 2 diabetes, cardiovascular disease, and several malignancies [14].

Circulating adiponectin exists as three oligomeric isoforms: low-molecular-weight (LMW) trimers, medium-molecular-weight (MMW) hexamers, and high-molecular-weight (HMW) multimers. Among these, HMW adiponectin is considered the most biologically active isoform and is the principal mediator of insulin sensitivity and glucose homeostasis [9]. Mutations affecting adiponectin multimerization reduce circulating HMW adiponectin concentrations and have been linked to insulin resistance and type 2 diabetes, suggesting that HMW adiponectin may be a more sensitive metabolic biomarker than total adiponectin [15]. Likewise, the HMW-to-total adiponectin (HMW: TA) ratio has been proposed as a superior indicator of insulin sensitivity and metabolic syndrome and has demonstrated stronger associations with disease severity compared to total adiponectin alone in patients with coronary artery disease, polycystic ovary syndrome, and late menopause [16,17,18,19,20,21]. For example, Wang et al. reported that both total and HMW adiponectin were inversely associated with triglyceride concentrations, whereas HMW adiponectin and the HMW:TA ratio were negatively correlated with low-density lipoprotein cholesterol and C-reactive protein [17]. Although HMW adiponectin appears to be the most metabolically active isoform, its specific role in AD remains incompletely understood because most clinical studies measure total circulating adiponectin rather than individual isoforms. Consequently, the question of whether total adiponectin, HMW adiponectin, or the HMW:TA ratio represents the most informative biomarker for AD requires further investigation.

Adipose tissue functions as an active endocrine organ composed of adipocytes, preadipocytes, macrophages, endothelial cells, fibroblasts, and immune cells that collectively regulate systemic energy balance and glucose homeostasis [22]. Through the secretion of adipokines, including adiponectin, adipose tissue communicates with multiple organs to coordinate lipid metabolism, insulin sensitivity, inflammation, and immune responses while also serving as the body’s principal energy reservoir [13]. Given these diverse physiological functions, disturbances in adiponectin signaling contribute to metabolic disorders, including obesity, insulin resistance, type 2 diabetes, atherosclerosis, and cardiovascular disease, all of which are recognized risk factors for neurodegenerative diseases such as AD and Parkinson’s disease (PD) [23,24].

Beyond its metabolic actions, adiponectin has emerged as an important regulator of central nervous system function. Experimental studies demonstrate that adiponectin suppresses microglial activation induced by amyloid-β oligomers, promotes hippocampal neural progenitor cell proliferation, and enhances hippocampal synaptic plasticity through activation of the AdipoR1/AMPK signaling pathway, thereby improving spatial learning and working memory in animal models [23,25]. Adiponectin also exerts anti-inflammatory and antioxidant effects through its receptors, AdipoR1 and AdipoR2, suggesting a potential role in regulating neuroinflammation [26,27,28]. Conversely, reduced adiponectin levels, commonly observed in obesity and diabetes, are associated with chronic low-grade systemic inflammation that may exacerbate neuroinflammatory processes. These findings support a biologically plausible role for adiponectin in neuroprotection; however, most evidence is derived from cellular and animal models, and its clinical significance in AD remains uncertain. Neuroinflammation contributes to neuronal dysfunction through increased production of reactive oxygen and nitrogen species, glial activation, and the accumulation of amyloid-β and tau pathology, ultimately promoting neurodegeneration [24,29]. Therefore, the modulation of adiponectin signaling represents a promising mechanistic pathway that warrants further investigation in human AD.

## 4. Adiponectin and BMI

Obesity is characterized by chronic low-grade inflammation and is consistently associated with reduced circulating adiponectin levels, insulin resistance, metabolic syndrome, and an increased risk of cardiovascular disease and type 2 diabetes [30,31]. These metabolic disorders are recognized as risk factors for cognitive decline and AD and may contribute indirectly through chronic inflammation, oxidative stress, and impaired glucose metabolism [32].

Body mass index (BMI), visceral adiposity, age-related weight loss, frailty, and metabolic status are important confounding factors that should be considered when interpreting circulating adiponectin concentrations in AD [33]. The relationship between adiponectin and BMI is complex and appears to differ across the lifespan. During midlife, increased BMI and visceral adiposity are generally associated with lower adiponectin concentrations, whereas weight loss and frailty, which commonly occur during the later stages of AD, have been associated with paradoxically elevated circulating adiponectin levels [34]. This phenomenon may partially explain the inconsistent associations reported between adiponectin and cognitive impairment in clinical studies and highlights the importance of considering body composition and nutritional status when interpreting circulating adiponectin concentrations in older adults.

Visceral adiposity appears to be a particularly important determinant of adiponectin concentrations. Gariballa et al. reported a significant inverse association between visceral fat mass and circulating adiponectin among overweight and obese adults [35]. Similarly, studies in South Asian populations demonstrated that reduced adiponectin levels were strongly associated with increased adiposity and insulin resistance, suggesting that ethnic differences in adipose tissue distribution and metabolic risk may influence circulating adiponectin concentrations [32]. These observations indicate that differences in BMI, visceral adiposity, ethnicity, and metabolic health should be carefully considered when comparing adiponectin levels across AD cohorts.

Collectively, current evidence suggests that circulating adiponectin reflects not only adipose tissue function but also changes in body composition, metabolic status, and aging. Consequently, BMI, visceral adiposity, late-life weight loss, and frailty should be considered major confounding variables in studies investigating the relationship between adiponectin and AD. Future longitudinal studies incorporating detailed assessments of body composition and metabolic health are needed to determine whether alterations in adiponectin represent a causal mechanism, a compensatory response, or a biomarker of disease progression.

## 5. Adiponectin and AD

Alzheimer’s disease is characterized by progressive cognitive decline and has been associated with impaired brain insulin signaling and reduced insulin sensitivity [36]. Although adiponectin is abundant in circulation, its concentration in the central nervous system is considerably lower [37,38]. Experimental studies have shown that plasma adiponectin levels are reduced in AD mouse models compared with wild-type mice [39,40]. Likewise, some observational studies have reported lower circulating adiponectin levels in women with AD, suggesting a possible association between adiponectin dysregulation and disease risk [41]. Experimental evidence further suggests that adiponectin may exert neuroprotective effects through the modulation of insulin signaling, neuroinflammation, and oxidative stress; however, these findings are derived primarily from cellular and animal models and have not been confirmed in adequately powered clinical trials in humans [42,43].

Human evidence remains inconsistent. Several cross-sectional studies have reported positive associations between circulating adiponectin concentrations and markers of AD pathology, including cerebrospinal fluid tau concentrations and measures of cognitive status, with some associations appearing stronger in specific subgroups, such as male APOE ε4 non-carriers and women [44,45]. In contrast, longitudinal cohort studies and Mendelian randomization analyses have generally failed to demonstrate a consistent causal relationship between circulating adiponectin levels and the future risk or progression of AD [46,47]. Collectively, these findings support the concept of the “adiponectin paradox,” in which elevated circulating adiponectin may reflect compensatory responses, frailty, weight loss, or other age-related metabolic changes rather than direct neuroprotection.

Among the circulating adiponectin isoforms, the HMW form is regarded as the biologically most active form and is most strongly associated with insulin sensitivity [9]. However, its relevance to AD has not been established because most clinical studies have measured total circulating adiponectin rather than individual isoforms. Consequently, it remains uncertain whether HMW adiponectin or the HMW-TA ratio provides greater predictive value for neurodegeneration. Future longitudinal studies integrating adiponectin isoforms with established AD biomarkers, including cerebrospinal fluid and neuroimaging measures, are needed to determine their potential clinical utility and to evaluate the proposed interaction among VD, L-Cys, and adiponectin.

## 6. Cross-Sectional Findings

Human studies investigating adiponectin in AD have yielded inconsistent results (Table 1). Several cross-sectional analyses report higher plasma or serum adiponectin in patients with AD compared to cognitively normal controls. In the ADNI1 cohort, elevated adiponectin was associated with worse cognition and increased CSF tau levels in APOE ε4 non-carrier males, although it did not predict longitudinal decline [46]. Another study found that serum adiponectin levels were 33% higher in AD than in mild cognitive impairment (MCI), with cerebrospinal fluid adiponectin correlating positively with Aβ_42_ and cognitive performance in women [48].

The studies summarized in Table 1, Tables 2 and 3 were selected to represent key clinical, epidemiological, mechanistic, and interventional evidence relevant to adiponectin biology, VD, L-Cys, and AD. Priority was given to landmark studies, recent high-quality investigations, randomized clinical trials, and representative observational studies that collectively illustrate the current state of evidence rather than providing an exhaustive literature listing.

Preclinical evidence mostly supports a protective role for adiponectin signaling in neuronal health; receptor agonists (e.g., AdipoRon) improve outcomes in animal models [53]. Prospective cohort data are less supportive. In a large population-based study with ~7 years of follow-up, baseline HMW adiponectin levels were not associated with incident dementia, including AD or vascular dementia [54]. Similarly, a Mendelian randomization study found no evidence for a causal protective effect of adiponectin on AD risk [55]. These discrepancies may reflect the adiponectin paradox. While experimental evidence supports protective signaling, elevated circulating adiponectin in older adults may instead mark late-life catabolism, weight loss, or systemic inflammation, the factors that accompany or precede dementia [33]. Thus, in humans, high adiponectin may correlate with disease severity or frailty rather than directly mediating protection. While observational human studies provide mixed evidence, mechanistic investigations offer clearer insights into adiponectin’s neurobiological roles.

The inconsistent associations reported between circulating adiponectin and AD likely reflect multiple interacting factors, including differences in age, sex, APOE genotype, body composition, frailty, weight loss, disease stage, adiponectin isoforms, and the biological specimen analyzed, such as plasma, serum, or cerebrospinal fluid. Methodological heterogeneity, including differences in study design, population characteristics, assay methodologies, and statistical adjustment for confounding variables, further contributes to these conflicting findings and likely underlies the adiponectin paradox [33,56].

## 7. Mechanistic Insights into Adiponectin in the Brain

### 7.1. Preclinical Mechanistic Evidence

Experimental studies consistently demonstrate that adiponectin exerts neuroprotective effects through multiple molecular pathways relevant to AD. In cellular and animal models, adiponectin enhances neuronal glucose metabolism and insulin signaling by activating the AMPK and PI3K/Akt pathways, thereby reducing tau hyperphosphorylation, improving synaptic plasticity, and enhancing learning and memory in AD-like mouse models [19,57]. Adiponectin also attenuates neuroinflammation by suppressing microglial activation and reducing the production of pro-inflammatory cytokines. Adiponectin-deficient mice exhibit exaggerated neuroinflammatory responses and accelerated AD-like pathology, whereas the restoration of adiponectin signaling promotes anti-inflammatory phenotypes and preserves blood–brain barrier integrity through the maintenance of tight junction proteins and modulation of amyloid-β (Aβ) transporters [57,58].

*In vitro* studies further demonstrate that adiponectin protects neurons against Aβ-induced oxidative stress, excitotoxicity, and apoptosis while stimulating autophagy–lysosomal pathways that facilitate the clearance of toxic protein aggregates. Consistent with these findings, adiponectin supplementation or pharmacological activation of adiponectin receptors reduces Aβ deposition and tau pathology in several AD mouse models [40,59]. Collectively, these studies provide strong biological plausibility that adiponectin signaling contributes to neuroprotection. However, these findings are derived primarily from experimental models and should not be interpreted as evidence of clinical efficacy in humans.

### 7.2. Translational Gaps and Future Directions

Despite compelling mechanistic evidence, several important translational gaps limit the interpretation of adiponectin as a biomarker or potential therapeutic target in AD. Human observational studies have produced inconsistent findings, with several cohorts reporting higher circulating adiponectin concentrations in individuals with cognitive impairment or AD, despite the neuroprotective effects observed in experimental models [12]. These apparently contradictory findings may reflect compensatory responses to neurodegeneration, age-related frailty, weight loss, systemic inflammation, or changes in body composition rather than a direct causal relationship with disease progression. Indeed, in several studies, the association between adiponectin and AD was attenuated after adjustment for age, sex, BMI, or APOE genotype, suggesting that peripheral adiponectin levels may be influenced by metabolic and demographic factors independent of brain pathology [52].

Additional limitations include the scarcity of well-powered prospective studies integrating repeated adiponectin measurements with amyloid and tau PET imaging, cerebrospinal fluid biomarkers, and longitudinal cognitive assessments, making it difficult to establish temporal or causal relationships [60]. Moreover, although experimental studies implicate AdipoR1/AdipoR2–AMPK signaling pathway in neuroprotection, little is known about adiponectin receptor expression, receptor sensitivity, or downstream signaling in the human AD brain, limiting mechanistic interpretation [61]. Finally, while recent meta-analyses demonstrate consistent benefits of adiponectin receptor agonists in experimental AD models, no clinical trials have evaluated adiponectin receptor activation in patients with mild cognitive impairment or AD [62]. Therefore, the therapeutic potential of adiponectin signaling remains hypothesis-generating and requires validation in well-designed longitudinal studies and randomized clinical trials.

## 8. Synergistic Effects of Vitamin D and L-Cysteine

Emerging evidence highlights a synergistic interaction between VD and L-Cys in regulating glutathione (GSH) and metabolic pathways. Co-supplementation with VD and L-Cys significantly enhances VD bioavailability and activity by improving GSH status, upregulating VD-regulatory genes, and promoting glucose metabolism [10,11,63].

Current evidence suggests that VD and L-Cys may influence biological pathways implicated in AD pathogenesis, including oxidative stress, inflammation, glutathione metabolism, and adiponectin signaling [4,64,65]. However, direct evidence demonstrating how these interventions prevent or modify AD in humans is currently lacking.

### 8.1. Vitamin D and Metabolic Regulation

VD is a fat-soluble hormone essential for calcium homeostasis, bone health, and immune regulation. VD receptors (VDRs) are expressed in pancreatic β-cells, adipocytes, skeletal muscle, and immune cells, suggesting that VD contributes to multiple metabolic pathways beyond bone metabolism. Experimental studies have demonstrated that the activation of the VDR improves insulin secretion, enhances insulin receptor expression, suppresses pro-inflammatory cytokine production, and reduces oxidative stress, thereby contributing to improved metabolic homeostasis [65]. Conversely, VD deficiency has been associated with obesity, insulin resistance, metabolic syndrome, and type 2 diabetes mellitus, conditions that are recognized risk factors for cognitive decline and AD [63,65,66,67].

Clinical studies conducted in individuals with obesity, metabolic syndrome, and type 2 diabetes have reported that VD supplementation can improve insulin sensitivity, glycemic control, and inflammatory status, although the magnitude of these effects varies among studies. Several randomized controlled trials and meta-analyses suggest modest improvements in fasting glucose, HOMA-IR, and circulating inflammatory biomarkers following VD supplementation, particularly in VD-deficient individuals. Nevertheless, the benefits appear more consistent for metabolic outcomes than for neurological endpoints. Therefore, although VD may indirectly influence pathways implicated in AD through improved metabolic regulation, evidence from metabolic populations should not be interpreted as demonstrating efficacy in preventing or treating neurodegenerative disease [68,69,70,71].

### 8.2. Vitamin D and Circulating Adiponectin

Emerging evidence suggests that VD may influence adipose tissue biology by regulating adipokine production, including adiponectin [65,67]. Experimental studies indicate that VD can modulate adipocyte differentiation, suppress inflammatory signaling within adipose tissue, and improve insulin sensitivity through the activation of VD receptor-mediated pathways [72]. Several clinical studies involving individuals with obesity, metabolic syndrome, and type 2 diabetes have reported modest increases in circulating adiponectin concentrations following VD supplementation, while the findings remain inconsistent across different populations (Table 2). Variability in study design, baseline VD status, obesity severity, and supplementation regimens likely contribute to these heterogeneous results [73,74].

### 8.3. Vitamin D in Older Adults, MCI, and AD

VD deficiency is highly prevalent among older adults and has been associated with cognitive impairment, frailty, and an increased risk of dementia in numerous observational studies. VD may influence brain health through several mechanisms, including the modulation of neuroinflammation, maintenance of calcium homeostasis, reduction in oxidative stress, regulation of neurotrophic factors, and enhancement of amyloid-β clearance [79,80]. VD receptors and the enzymes responsible for VD metabolism are widely expressed throughout the central nervous system, supporting a potential role for VD in neuronal survival and cognitive function [81].

Despite these biological observations, clinical evidence supporting VD supplementation for the prevention or treatment of AD remains inconclusive. Several prospective cohort studies have reported an association between low serum 25-hydroxyvitamin D concentrations and increased risk of cognitive decline or dementia [82]. However, randomized controlled trials conducted in older adults and individuals with MCI have generally produced inconsistent findings, with many studies showing limited or no significant improvement in cognitive performance following VD supplementation (Table 3). These discrepancies likely reflect differences in study populations, baseline VD status, supplementation dose and duration, cognitive assessments, and disease stage. Consequently, current evidence does not support VD supplementation as an established therapy for AD, although maintaining adequate VD status remains important for overall health [70,71,81,82].

Although the relationship between VD and adiponectin has been extensively investigated in metabolic disorders, relatively few studies have evaluated this interaction in older adults or individuals with cognitive impairment [69,70,71,81]. Moreover, most clinical trials examining VD supplementation in populations at risk for AD have not measured circulating adiponectin as an outcome. Consequently, it remains unclear whether VD-mediated modulation of adiponectin contributes to neuroprotection or influences AD progression. At present, the proposed interaction between VD, adiponectin signaling, and AD should therefore be regarded as biologically plausible but largely hypothesis-generating, requiring validation in longitudinal cohort studies and randomized clinical trials incorporating both metabolic and neurodegenerative biomarkers [47,83,84].

Collectively, evidence from metabolic disorders, aging populations, and adiponectin-related studies supports a biological interaction between VD, metabolic homeostasis, and inflammatory regulation [85,86]. However, these findings originate from distinct clinical contexts and should not be considered interchangeable. While VD supplementation demonstrates the most consistent benefits in improving metabolic and inflammatory parameters, direct evidence linking VD-induced adiponectin modulation to improved AD biomarkers or cognitive outcomes is currently lacking [87]. Future prospective studies and randomized clinical trials should simultaneously assess VD status, adiponectin isoforms, and validated AD biomarkers to determine whether this proposed mechanistic pathway has translational relevance in neurodegenerative disease.

## 9. L-Cysteine and Redox Regulation

L-Cys, a semi-essential amino acid, plays a critical role in protein structure through disulfide bond formation and serves as a precursor for GSH, a master antioxidant [88]. By supporting GSH synthesis, L-Cys supplementation enhances redox balance, reduces oxidative stress, and mitigates inflammation [89]. Dietary supplementation with L-Cys can enhance glutathione synthesis when it is impaired, thereby improving redox balance and alleviating oxidative stress. The removal of free radicals may confer additional advantages, such as shorter recovery times after certain surgical interventions [88]. Furthermore, the antioxidant properties of L-Cys are associated with a reduced risk of cerebrovascular accidents and a reduced risk of noise-induced hearing loss [90]. A deficiency in L-Cys or GSH exacerbates ROS accumulation, impairing cellular homeostasis and contributing to metabolic and neurodegenerative diseases. Clinical and preclinical studies highlight the therapeutic potential of L-Cys supplementation in restoring GSH levels, improving mitochondrial function, and reducing oxidative stress-related damage [91,92]. Increased GSH reduces oxidative stress in adipocytes and systemic tissues, which can restore normal adipokine secretion patterns, including adiponectin [9]. In adipocytes, L-Cys supplementation upregulated DsbA-L (disulfide bond A-like protein), a chaperone important for adiponectin multimer assembly and secretion, thereby increasing adiponectin production [65].

L-Cys and *N*-acetylcysteine (NAC) are related sulfur-containing compounds but should not be considered equivalent interventions. L-Cys is a naturally occurring amino acid that serves as a direct precursor for glutathione synthesis, whereas NAC is a pharmacological derivative with improved stability and bioavailability that is converted to cysteine after administration [93]. Consequently, findings from NAC studies should not be directly extrapolated to L-Cys supplementation without appropriate qualification. NAC lowers pro-inflammatory cytokines (MCP-1) and normalizes inflammatory signaling, both of which promote adiponectin expression and protect neurons from inflammation-driven damage [94]. NAC or cysteine-containing compounds protect against diet- or toxin-induced hippocampal/metabolic changes and improve memory in some AD-model or high-fat-diet animal studies. These often show concurrent improvements in oxidative stress and adipokine profiles [95]. Randomized clinical trials evaluating NAC have been conducted in individuals with metabolic syndrome or obesity rather than in older adults or patients with AD. These studies generally demonstrate improvements in oxidative stress, inflammatory markers, insulin resistance, and glycemic control; however, effects on circulating adiponectin have been inconsistent. Consequently, current evidence does not support the conclusion that NAC consistently increases circulating adiponectin in humans, and no comparable randomized trials have been conducted in MCI or AD populations [96].

Human randomized clinical trials have demonstrated that VD supplementation and selected cysteine-related interventions can improve metabolic biomarkers and, in some studies, increase circulating adiponectin concentrations in individuals with obesity or metabolic disorders [11]. In contrast, much of the mechanistic evidence supporting adiponectin upregulation by L-Cys or NAC derives from cell culture and animal models. These experimental findings provide biological plausibility but should not be interpreted as evidence of clinical efficacy in AD (Table 4). Reviews of adiponectin in AD highlight not only its neuroprotective potential but also the “adiponectin paradox” in humans [38].

Studies have shown that L-Cys supplementation increased the secretion of adiponectin by inhibiting ROS and MCP-1 and increasing glucose utilization in adipocytes [9,65]. L-Cys increases adiponectin levels in cells, animals, and some human metabolic studies and has protective antioxidant/anti-inflammatory effects that could be beneficial in AD. However, no direct human AD trials have shown that cysteine-driven adiponectin changes reduce AD pathology or clinical decline.

## 10. Vitamin D + L-Cysteine: A Hypothesis-Generating Framework

Experimental studies have suggested a potential interaction between VD and L-Cys in regulating GSH metabolism and related metabolic pathways. Preclinical investigations indicate that co-supplementation with VD and L-Cys may improve GSH status, influence the expression of VD-related genes, and enhance glucose metabolism more effectively than VD alone [10,11]. Clinical studies conducted in individuals with metabolic disorders have also reported improvements in oxidative stress and selected metabolic biomarkers following VD and cysteine-related interventions. However, these studies were not conducted in populations with mild cognitive impairment or AD, and whether these metabolic effects translate into clinically meaningful neuroprotective benefits remains unknown [11,64,103].

**Figure 1:**
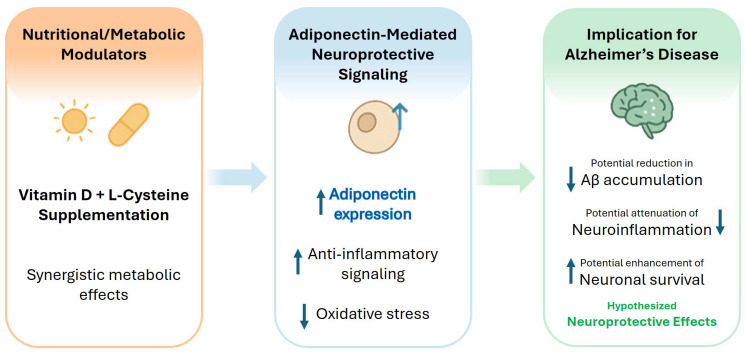
Proposed hypothetical model illustrating how VD and L-Cys may influence adiponectin signaling, oxidative stress, and inflammatory pathways implicated in AD. The downstream neuroprotective effects depicted are based primarily on experimental and mechanistic evidence. Direct clinical evidence demonstrating that combined VD and L-Cys supplementation improves AD biomarkers or cognitive outcomes through adiponectin signaling is currently lacking.

Adiponectin and its regulatory pathways represent biologically plausible targets for further investigation into metabolic and neurodegenerative diseases. Experimental studies suggest that interventions capable of increasing adiponectin concentrations, modifying adiponectin isoforms, or activating AdipoR1/AdipoR2 signaling may influence metabolic regulation and neuroinflammatory pathways. Similarly, strategies aimed at improving redox homeostasis through VD and L-Cys supplementation may indirectly modulate adiponectin-related pathways and insulin sensitivity. Nevertheless, evidence supporting these mechanisms is derived largely from experimental models and metabolic populations, and direct clinical evidence demonstrating therapeutic benefit in AD is currently lacking. Therefore, these observations should be regarded as hypothesis-generating rather than evidence of an established therapeutic approach. Well-designed longitudinal studies and randomized clinical trials involving older adults, individuals with mild cognitive impairment, and patients with AD are needed to determine whether the modulation of VD, L-Cys, and adiponectin influences disease biomarkers or clinical outcomes (Figure 1).

## 11. Conclusions

Adiponectin represents a key integrative link between metabolic homeostasis and neuroprotection. Its roles in glucose regulation, lipid metabolism, anti-inflammatory signaling, and neuronal health highlight its therapeutic potential. In summary, current evidence provides robust biological plausibility and substantial experimental support for interactions among VD, L-Cys, adiponectin, oxidative stress, and neuroinflammation. Human studies investigating circulating adiponectin in AD have yielded mixed findings, likely reflecting heterogeneity in study populations, adiponectin isoforms, disease stage, and metabolic status. Clinical evidence supporting VD and L-Cys supplementation is strongest for improving metabolic and redox homeostasis, whereas direct evidence linking these interventions to the modulation of adiponectin signaling and improved AD outcomes is currently lacking. Future mechanistic studies, translational investigations, and adequately powered randomized clinical trials are required to determine whether the proposed interactions among VD, L-Cys, and adiponectin influence Alzheimer’s disease biomarkers or clinical outcomes.

## Figures and Tables

**Table 1:** Key studies on adiponectin and Alzheimer’s disease.

Study	Design/Population	Key Adiponectin Finding	Outcome/Interpretation
Une et al., 2010 [47]	Cross-sectional, 18 MCI, 27 AD patients, and 28 controls	Plasma and CSF adiponectin positively correlated; plasma APN was higher in MCI and AD	Higher plasma APN levels in MCI and AD may be due to weight loss, decrease in fat tissue, and appetite change, but a small sample size limits strong conclusion
Letra et al., 2019 [48]	Cross-sectional; 71 MCI vs. 53 AD patients	Serum adiponectin is ~33% higher in AD vs. MCI	Higher serum adiponectin in AD may reflect compensatory mechanisms; associations differ by sex; limited predictive power (AUC modest)
Kim et al., 2022 [49]	Longitudinal cohort: 156 amyloid-positive MCI individuals	Higher plasma adiponectin predicted faster cognitive decline	Suggests adiponectin may be a prognostic biomarker for progression in Aβ(+) MCI
Mooldijk et al., 2022 [50]	Prospective population cohort; 177 dementia patients and 945 controls	APN associations varied depending on covariates	The relationship between APN and dementia risk is complex and context-dependent
Carbone et al., 2024 [51]	Longitudinal study; 396 MCI, 112 AD patients and 58 controls	APN is linked with certain vascular/MRI outcomes, but not consistently with cognition	Adiponectin may reflect metabolic/vascular processes in AD
Sindzingre et al., 2025 [52]	Case–control; 142 AD patients and 64 neurological controls	AD patients had higher plasma APN, but explained by age, sex, and BMI	APN difference disappears after adjustment, likely confounded by frailty factors

Aβ, amyloid beta; AD, Alzheimer’s disease; APN, adiponectin; CSF, cerebrospinal fluid; MCI, mild cognitive impairment.

**Table 2:** Selected randomized clinical trials on VD supplementation and their effects on adiponectin levels.

Study	Population/Design	VD Intervention	Adiponectin Change/Effect Size
Baziar et al., 2014 [75]	81 T2D patients	50,000 IU VD/week for 8 weeks	No significant changes
Mohammadi et al., 2016 [76]	53 first-degree relatives of patients with T2D	50,000 IU VD/week for 12 weeks	VD status improved but did not significantly alter adiponectin levels
Mai et al., 2017 [74]	24 obese patients	600,000 IU of cholecalciferol for 4 weeks	Significant increase in HMW-A expression
Seyyed Abootorabi et al., 2018 [77]	44 women with PCOS	50,000 IU of oral VD3/week for 8 weeks	Significant increase in adiponectin, fasting glucose, and β-cell function
Hosseinzadeh et al., 2020 [78]	45 pregnant women with GDM	Single dose of VD injection (300,000 IU)	Adiponectin levels increased significantly after VD injection (*p* = 0.01)
Mousa et al., 2020 [67]	54 overweight/obese, VD-deficient adults	Single bolus of 100,000 IU + 4000 IU/day cholecalciferol for 16 weeks vs. placebo	Adjusted increase in adiponectin: β = 13.7 ng/mL (95% CI: 2.0 to 25.5; *p* = 0.02) after adjusting for baseline, season, sun exposure, diet, age, sex, and % body fat
Rashidmayvan et al., 2023 [68]	79 patients with metabolic syndrome, double-blind RCT	Fortified milk containing 1500 IU VD for 10 weeks vs. non-fortified milk	Serum adiponectin significantly increased in the VD-fortified milk group compared to the control (*p* = 0.034)
Schmitt et al., 2023 [66]	160 postmenopausal women, RCT	1000 IU cholecalciferol (VD) daily for 9 months vs. placebo	+18.6% increase in adiponectin in the VD group; absolute levels: VD group ~18.5 ng/mL vs. placebo ~11.5 ng/mL at endpoint (*p* = 0.047)

HMW-A, high-molecular-weight adiponectin; GDM, gestational diabetes mellitus; T2D, type 2 diabetic patients; PCOS, polycystic ovary syndrome.

**Table 3:** Randomized clinical trials of VD in older/dementia-risk populations.

Study	Population/Design	VD Intervention	Outcomes	Adiponectin Measured?
Jia et al., 2019 [69]	210 elderly AD patients, 12-month RCT	800 IU/day	VD supplementation improved cognitive function and decreased Aβ-related biomarkers	No—measured only Aβ Biomarkers (Aβ42, APP, BACE1, APP mRNA, BACE1 mRNA), not adiponectin
Yang et al., 2020 [81]	183 older adults (≥65) with MCI, 12-month RCT	800 IU/day	Cognitive tests, telomere length, oxidative stress markers	No—no adiponectin reported
Montero-Odasso M et al., 2023 [70]	175 older adults (65–84) with MCI, multidomain RCT	VD: 10,000 IU, 3×/week, plus exercise and cognitive training in some arms	VD supplementation had no significant effect on ADAS-Cog, cognition	No—focus on cognition; adiponectin not reported
Lonnroos et al., 2025 [71]	~2492 older adults, 5-year RCT	1600 IU/day or 3200 IU/day vs. placebo	Incident dementia (registry-based)	No—they did not measure adiponectin

Aβ, amyloid beta; APP, amyloid precursor protein; BACE1, beta-secretase 1; MCI, Mild Cognitive Impairment; ADAS-Cog, Alzheimer’s Disease Assessment Scale–Cognitive Subscale.

**Table 4:** Primary studies showing effects of L-Cys and NAC on adiponectin and/or its related signaling.

Study (Year)	Design/Population	Key Adiponectin Finding
Achari AE, Jain SK, 2016 [65]	3T3-L1 adipocytes (high glucose)	LC (250–500 µM) increased DsbA-L protein and total and HMW adiponectin secretion.
Achari AE, Jain SK. 2017 [9]	3T3-L1 adipocytes + insulin + high glucose	LC + insulin dramatically boosted both total adiponectin and HMW adiponectin compared to either alone; also, ↑ GSH, GLUT-4.
Araki S, Dobashi K et al., 2006 [97]	3T3-L1 adipocytes treated with TNF-α	NAC (5–20 mM) prevented TNF-α-induced drop in adiponectin secretion; blocked NF-κB activation.
Calzadilla P et al., 2011 [98]	3T3-L1 preadipocytes differentiating to adipocytes	NAC inhibited differentiation markers (PPARγ, C/EBPβ), suggesting effects on adipocyte development, which could influence adiponectin output.
Ma Y et al., 2016 [99]	Mouse (C57BL/6) on high-fat diet + NAC in drinking water	NAC significantly increased adiponectin gene expression in adipose tissue, reduced insulin resistance and inflammation.
Raffaele M et al., 2018 [100]	3T3-L1 adipocytes/adipogenesis model	NAC treatment increased adiponectin expression, along with other metabolic regulators (DGAT1, FABP4).
Berry A et al., 2018 [101]	HFD mice treated with NAC	NAC supplementation increased adiponectin levels in males (but not in females) per tissue/serum measurements.
Panahi et al., 2022 [96]	76 people with metabolic syndrome	NAC supplementation did not significantly increase the adiponectin levels
Balagopal et al., 2024 [102]	13 children with MASLD	NAC supplementation (600 or 1200 mg/day) did not significantly increase the adiponectin levels

HMW, high molecular weight; GSH, glutathione; GLUT-4, Glucose Transporter Type 4; NAC, *N*-acetylcysteine; TNF-α, Tumor necrosis factor-alpha; PPARγ, Peroxisome Proliferator-Activated Receptor gamma; C/EBPβ, CCAAT/enhancer-binding protein beta; MASLD, metabolic dysfunction-associated steatotic liver disease.

## Data Availability

No new data were created or analyzed in this study. Data sharing is not applicable to this article.
